# Identifying Child Anxiety Through Schools-identification to intervention (iCATS-i2i): protocol for single-arm feasibility trial

**DOI:** 10.1186/s40814-022-01140-x

**Published:** 2022-08-10

**Authors:** Tessa Reardon, Susan Ball, Maria Breen, Paul Brown, Emily Day, Tamsin Ford, Alastair Gray, Iheoma Green, Claire Hill, Bec Jasper, Thomas King, Michael Larkin, Ian Macdonald, Fran Morgan, Jack Pollard, Michelle Sancho, Falko F. Sniehotta, Susan H. Spence, Paul Stallard, Jason Stainer, Obioha C. Ukoumunne, Mara Violato, Chloe Williams, Victoria Williamson, Cathy Creswell

**Affiliations:** 1 Departments of Experimental Psychology and Psychiatry, University of Oxford, Oxford, UK; 2 NIHR ARC South West Peninsula (PenARC), University of Exeter, Exeter, UK; 3 Thames Valley Clinical Trials Unit, University of Reading, Reading, UK; 4 Bransgore C of E Primary School, Christchurch, UK; 5 Oxford NHS Foundation Trust, Oxford, UK; 6 University of Cambridge and Cambridge and Peterborough Foundation Trust, Cambridge, UK; 7 Health Economics Research Centre, Nuffield Department of Population Health, University of Oxford, Oxford, UK; 8 School of Psychology & Clinical Language Sciences, University of Reading, Reading, UK; 9 Parents and Carers Together, Suffolk, UK; 10 Life and Health Sciences, Aston University, Birmingham, UK; 11 Charlie Waller Trust, Thatcham, UK; 12 Square Peg, East Sussex, UK; 13 West Berkshire Council, Newbury, UK; 14 NIHR Policy Research Unit Behavioural Science, Newcastle University, Newcastle upon Tyne, UK; 15 School of Applied Psychology and Australian Institute of Suicide Research and Prevention, Griffith University, Brisbane, Australia; 16 Department of Health, University of Bath, Bath, UK; 17 Stanley Primary School, Strathmore Road, London, UK; 18 Institute of Psychiatry, Psychology and Neuroscience, King’s College London, London, UK

**Keywords:** Anxiety, Children, Screening, Schools, Identification, Early intervention, Online intervention, Parent-led intervention

## Abstract

**Background:**

Anxiety disorders are common among primary-school aged children, but few affected children receive evidence-based treatment. Identifying and supporting children who experience anxiety problems through schools would address substantial treatment access barriers that families and school staff often face. We have worked with families and school staff to co-design procedures that incorporate screening, feedback for parents, and the offer of a brief intervention in primary schools. This study sets out to assess the feasibility of a subsequent school-based cluster randomised controlled trial to evaluate these procedures. Our objectives are to ensure our procedures for identifying and supporting children with anxiety difficulties through primary schools are acceptable and there are no negative impacts, to estimate recruitment and retention rates, and to identify any changes needed to study procedures or measures.

**Methods:**

We will recruit six primary/junior schools in England (2 classes per school), and invite all children (aged 8–9) (*n* = 360) and their parent/carer and class teacher in participating classes to take part. Children, parents and class teachers will complete questionnaires at baseline and 12-week follow-up. Children who ‘screen positive’ on a 2-item parent-report child anxiety screen at baseline will be the target population (expected *n* = 43). Parents receive feedback on screening questionnaire responses, and where the child screens positive the family is offered support (OSI: Online Support and Intervention for child anxiety). OSI is a brief, parent-led online intervention, supported by short telephone sessions with a Children’s Wellbeing Practitioner. Participants’ experiences of study procedures will be assessed through qualitative interviews/discussion groups.

**Discussion:**

Evidence-based procedures for identifying and supporting children with anxiety difficulties through primary schools would improve children’s access to timely, effective intervention for anxiety difficulties.

**Trial registration:**

ISRCTN registry: ISRCTN30032471. Retrospectively registered on 18 May 2021.

**Supplementary Information:**

The online version contains supplementary material available at 10.1186/s40814-022-01140-x.

## Background

Anxiety disorders are the most common mental health disorder experienced by children and young people [1], and typically first emerge before a child reaches secondary school [2]. Anxiety disorders in childhood have a negative impact on social, academic and family functioning, and are associated with substantial societal burden [3]. Compared to the general population, children with anxiety disorders are at increased risk for ongoing anxiety difficulties, other mental health disorders, and reduced quality of life in adulthood [4, 5]. Children, families, and wider society would therefore benefit from effective early intervention for childhood anxiety disorders.

Psychological interventions are effective at treating anxiety disorders in children [6]. Evidence-based interventions typically draw on Cognitive Behavioural Therapy (CBT) principles and can be delivered using varied formats (e.g., in groups or one-to-one; face-to-face or online; therapists working with children and/or parents) and amount of therapist-support, with no consistent differences in child outcomes according to delivery format or amount of therapist contact time [7]. Despite this evidence-base, very few children who experience anxiety disorders access such interventions. A UK survey of parents of children (aged 7–11) with anxiety disorders identified through schools found that only 2% had received CBT [8]. Families report considerable barriers to seeking support for child anxiety problems, including difficulties determining whether or not their child’s anxiety warrants concern, and how and when to seek help [8, 9]. Where families do contact professionals available support is limited, with long waiting lists to access specialist services, and families describe that their concerns are often dismissed, with little support and guidance provided on how best to manage a child’s anxiety problems as a family [8, 9]. School staff are often the first point of contact for families with concerns about a child’s mental health, but feel ill-equipped to identify and support children with common mental health problems [10]. Universal screening in schools has the potential to address identification barriers, but is only recommended if suitable screening tools are available and evidence-based intervention and support is offered where difficulties are identified [11, 12]. Currently, there is no established approach to identifying and supporting children with anxiety problems that incorporates both universal screening and delivery of evidence-based interventions through primary schools.

In a previous study, we used a co-design approach, and worked with children, parents, school staff, and other key stakeholders to develop procedures for identifying and supporting children with anxiety problems through primary schools (Identifying Children Through Schools-identification to intervention; iCATS-i2i [13]). The co-designed iCATS-i2i procedures incorporate screening, written and telephone feedback for parents and the offer of a brief evidence-based intervention. The intervention is an online version of an effective and cost-effective parent-led treatment for child anxiety disorders [14–16] (OSI: Online Support and Intervention for child anxiety). Delivering support online and directly to parents offers the potential to maximise efficiency and provide families with the skills and confidence to manage a child’s anxiety problems as a family. In-depth interviews informed the development of an initial iteration of the iCATS-i2i procedures which we then delivered in three primary schools to collect feedback from participants on their experiences, and used this feedback to further refine and finalise procedures. In parallel to this co-design work, we also sought to identify short questionnaire measures (child, parent, and/or teacher report) that are quick and easy to administer and able to discriminate between children with and without anxiety disorders with a sufficient level of accuracy to use for screening purposes [17]. Cut-off scores on parent-report questionnaires consisting of 2 to 9 items were able to identify children with anxiety disorders with a reasonable level of accuracy (75–76% sensitivity and 73–82% specificity), but neither child nor teacher report questionnaires were able to achieve > 70% sensitivity and specificity. As the 2-item parent-report measure achieved comparable accuracy to longer parent-report measures, we prioritised brevity and have now incorporated this 2-item measure into the iCATS-i2i procedures as a screening tool to identify families to offer support. Completing screening questionnaires and receiving feedback on responses can help address identification barriers, but as this screen will miss some children who may benefit (false negatives), OSI will also be made available to other families who feel they would benefit, regardless of screening outcomes. Although our measurement study findings indicated child and teacher-reports were not sufficiently accurate to use for screening purposes, our co-design work identified the importance of involving children and class teachers in the screening process, and therefore child-, teacher-, and parent-report questionnaires are collected as part of the iCATS-i2i procedures. In order to feel confident we can progress to a definitive randomised controlled trial to evaluate the effectiveness and cost-effectiveness of the iCATS-i2i procedures, in the current study we now set out to test the feasibility of these co-designed procedures, incorporating our brief parent-report screening tool.

### Aims and objectives

This study aims to assess the feasibility of progressing to a subsequent cluster randomised controlled trial to evaluate our co-designed procedures for identifying and supporting children (aged 8–9) with anxiety difficulties through primary schools. Our objectives are to establish (i) whether there are any negative impacts of study procedures, (ii) any concerns about the acceptability of the study procedures, (iii) whether target recruitment and retention rates are feasible, (iv) whether the proposed clinical and health economic measures capture all the relevant information and outcomes, and (v) any changes needed to study procedures or outcome measures. We will use a single-arm design to efficiently evaluate the feasibility of study procedures, prior to progressing to a definitive randomised controlled trial with an inbuilt pilot phase.

## Methods

### Design

This is a single-arm feasibility trial and will follow the SPIRIT [18] recommendations and reporting guidance (see Additional file 1 for SPIRIT checklist). Children (aged 8–9) from six primary/junior schools, their parent/carer and class teacher will complete questionnaire measures at baseline and 12-week follow-up. The baseline assessment includes a parent-report 2-item (each item scored 0–3) child anxiety screening questionnaire, and children who screen positive on this questionnaire (score ≥ 3 out of 6) will be the target population. As long as baseline measures are collected from at least one reporter (child, teacher, parent), parents/carers will receive written feedback. Where parents/carers completed the screening questionnaire, this will include feedback on whether responses indicate their child may be experiencing difficulties with anxiety (screen positive) or is unlikely to be experiencing difficulties with anxiety (screen negative). If the child screens positive, parents/carers will be invited to a feedback telephone call with a Children’s Wellbeing Practitioner (CWP) and offered a brief, parent-led online intervention (OSI: Online Support and Intervention for child anxiety). OSI will also be made available to all parents/carers who express an interest, regardless of screening outcomes, and schools will be provided with materials for a whole-class lesson on managing everyday fears and worries that can be facilitated by the study team and/or school staff. Qualitative interviews and/or discussion groups will be conducted with a subsample of children and parents/carers, and staff working in or linked to participating schools. Recruitment and data collection will take place from November 2020 to September 2021.

### Setting

Participants will be recruited through six mainstream primary/junior schools in England. Schools need to have at least two year 4 classes (children aged 8–9 years) and a minimum of 40 pupils in year 4. Two classes per school will participate in the study.

### Participants

Inclusion criteria for the feasibility trial are as follows:

#### Children

- Child is in year 4 (aged 8–9 years) in a participating class, their parent/carer does not opt-out, and child provides assent.
- Child has sufficient English to give assent and complete questionnaires, with assistance if necessary.

#### Parents

- Parent/carer of child in year 4 in a participating class, and they provide consent. Where a parent/carer has more than one eligible child, they will be invited to consent/participate for each child.
- Parent/carer has sufficient English to give consent and to complete questionnaires, with assistance if necessary.

#### Class teachers

- Class teacher of participating child or nominated member of support staff who works regularly with the child.

The target population are children who screen positive (score ≥ 3 out of 6 on parent-report 2-item child anxiety questionnaire) at baseline.

Inclusion criteria for qualitative interviews/discussion groups are as follows:

#### Children

- Child is in a participating year 4 class, their parent/carer provides consent, they provide assent, and they have sufficient spoken English to take part in the interview/discussion, with assistance if necessary.

#### Parents

- Parent/carer of a child in a participating year 4 class, they provide consent, and they have sufficient spoken English to take part in the interview/discussion, with assistance if necessary.

#### School staff

- Member of staff or governor in a participating school, or a representative of another key stakeholder organisation with a professional role within or related to a participating school (e.g., a mental health service provider within a participating school).

### Recruitment

#### School recruitment

We aim to recruit six primary/junior schools that vary in relation to: geographic area, size of school, percentage of pupils eligible for free school meals, percentage of pupils on special educational needs support, percentage of pupils with English as an additional language. We will disseminate information about the study via our existing networks and social media adverts and contact individual schools via email and follow-up telephone calls. To help ensure recruited schools have varied geographic and demographic characteristics, we will record characteristics of eligible schools that express an interest and target particular schools as needed.

Written consent for the school’s participation (online or on paper) will be obtained from school headteachers, and each school will be asked to nominate an iCATs-i2i lead to act as the primary point of contact for the study team and co-ordinate study procedures. Where schools have more than two Year 4 classes, we will select two classes to participate.

#### Participant recruitment

Study information will be distributed to all children, parents, and class teachers in participating classes. We will work with each school to develop strategies to distribute study information and promote participation (e.g. distributing paper, electronic and/or video versions of study adverts and study information, advertising the study in school newsletters/websites, sending reminders via email and SMS). Where COVID-19 government and school guidance allow, researchers will run information sessions for parents, children and school staff at the school, and where this is not possible we will offer to run online information sessions and/or provide school staff with materials to facilitate these sessions themselves.

Parents will be given the opportunity to opt their child out of the study, and the school iCATS-i2i lead will keep a record of these children’s names and no information or data will be collected about or from these children. With the exception of any children whose parent opts out, all children in participating classes, and their parents and class teachers will be invited to participate and complete baseline questionnaires. Prior to participating, written assent (on paper or online) will be obtained from children, and parents and class teachers will provide written consent (on paper or online) prior to completing baseline measures and/or providing any information or data themselves. Explicit consent for audio recording will be required prior to parent/school staff qualitative interviews, and both parental consent and child assent will be obtained prior to qualitative interviews with children.

### Procedures

**Fig. 1:**
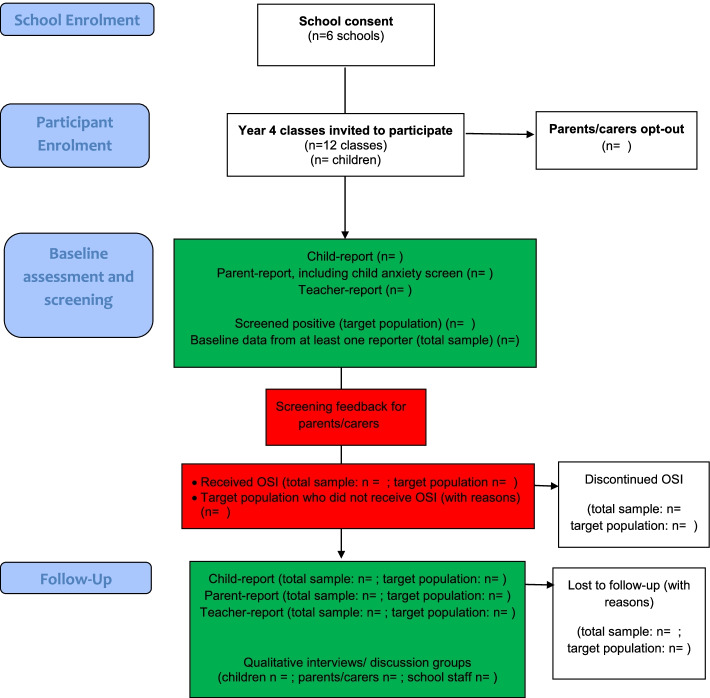
Overview of iCATS-i2i study procedures

An overview of study procedures and assessments are provided in Fig. 1 and Additional file 2.

Where possible, researchers will visit schools to administer baseline questionnaires (online or on paper) with groups of children, and children will be able to choose to complete the questionnaires at home if they prefer. If COVID-19 government or school guidance prohibits researchers from visiting schools and/or schools are closed for all or some children, we will work with schools to adapt this procedure (e.g. school staff administer questionnaires with children at school, some or all children complete questionnaires at home).

After administering the child baseline questionnaires, we plan to facilitate a lesson on managing everyday fears and worries for participating classes. Our intention is for researchers to lead this lesson with school staff support, on the same day or soon after children complete baseline questionnaires. However, we will be flexible in how and when we use this lesson to accommodate school staff preferences and potential COVID-19 restrictions, and will offer alternatives as needed (e.g. we provide school staff with the lesson materials to deliver themselves at a suitable time, we adapt materials to deliver an online lesson).

After baseline questionnaires are collected from children, schools will distribute consent and questionnaires to parents/carers, either on paper and/or online. Parents will be provided with an envelope to return paper questionnaires to school, ready for collection by the study team. In parallel, we will ask class teachers to complete teacher-report questionnaires (online or on paper) about all children in their class (where parents did not opt-out) and we will provide the iCATS-i2i school lead with forms to complete demographic, attendance, punctuality and learning information about these children, and to keep a record of school staff time spent on iCATS-i2i activities.

Where baseline questionnaires for a child are completed by at least one reporter (child, teacher, parent), the parent/carer will receive a feedback letter. Where parents provided us with their contact details, we will send this letter to them directly by post, and where we do not have parent contact details, we will provide schools with the letter in a sealed envelope to give to the parent or send home with the child. If parents complete the baseline questionnaires, including the brief child anxiety screen, the letter will provide feedback on whether the responses indicate the child may be experiencing difficulties with anxiety (screen positive) or is unlikely to be experiencing difficulties with anxiety (screen negative). Where the child screens positive, the letter explains that the study team will be in touch to arrange a feedback telephone call with the study Children’s Wellbeing Practitioner to discuss their questionnaire responses and offer the family OSI. Where the child screens negative or where there is no screening outcome (because the parent did not complete the screening questionnaire), the letter explains that OSI is available to all families who feel they may benefit and parents are invited to get in touch with the study team if they wish to discuss this further. A brief description of OSI and overview of sessions will be included with all parent feedback letters. With parental consent, the study team will share screening outcomes with the school iCATS lead.

Researchers will contact parents of children who screen positive by email, SMS, and/or telephone to arrange a convenient time for the feedback call. During the call, the CWP will invite parents to take part in OSI, and if parents agree, they will be given access to the online intervention and their telephone support sessions will be booked. If a parent who has not previously provided consent/completed parent-report baseline questionnaires contacts us following the feedback letter, and subsequently verbally agrees to take part in OSI, they will be asked to provide written consent and complete baseline questionnaires before starting the intervention. The CWP/s and their supervisor/s will complete a log throughout the intervention delivery, to record time spent on activities related to delivering and supervising OSI.

We will collect follow-up measures approximately 12-weeks after baseline questionnaires. As our primary aim is to assess the feasibility of collecting follow-up questionnaires and estimate retention rates, we will employ some flexibility with the exact timing of the follow-up assessment if COVID-19 restrictions/disruptions present particular barriers to timely data collection. Although a subsequent cluster randomised controlled trial would include follow-up assessments at 6, 12, and 24 months, retention rates immediately post-intervention and at 30-month follow-up were fairly similar in a recent primary school UK trial [19], and a shorter follow-up period will allow more timely progression to the main trial, if indicated.

All participants (children, parents, teachers) who complete baseline assessments will be asked to complete follow-up questionnaires. Follow-up questionnaires will be collected from children, and their parent and class teacher in parallel. Researchers will administer follow-up questionnaires (online or paper) with children at school, but we will adapt this procedure as needed in response to COVID-19 restrictions, as per at baseline. We will send parents and teachers personalised links to online follow-up questionnaires and/or provide the school iCATS-i2i lead with paper questionnaires to distribute to parents and teachers, together with envelopes to return to school for the study team to collect.

One-to-one interviews and/or discussion groups will be conducted with subgroups of children, parents, and school staff about their experiences of iCATS-i2i procedures during and after the intervention delivery phase. We anticipate that approximately 12 children and 12 parents will take part in an interview/discussion group, including children/parents where the child screened positive and screened negative, and those who took part in OSI and those who did not. We expect up to 10 members of school staff will take part in an interview/discussion group, and we will seek to include staff from each participating school, with varying roles (e.g. class teacher, iCATS-i2i lead, headteacher). Where appropriate, we will adopt further purposive sampling in order to learn from the experiences of participants who can offer a range of perspectives. For example, we will seek to include families where the child’s screening outcome may reflect a ‘false positive’ or ‘false negative’ by inviting parents/children where the anxiety screen score was just above and just below the cut-off. Interviews/discussion groups will be conducted either in-person at participating schools, by telephone or online video-call.

As a thank you, families will be offered a £10 gift voucher for each complete set of child/parent questionnaires and qualitative interview, and schools will be offered £200 for time spent on all study activities.

### Intervention

#### OSI

Parents work through a series of seven online modules which include simple text, audio versions of text, videos and animations, interactive activities, and inbuilt questionnaire measures. Online modules are released weekly for parents to complete in their own time. Each module takes about 20–30 min to complete and is supported by a brief telephone session with a CWP (approximately 20 min) once a week for 7 weeks, and a follow-up telephone session 4 weeks later. Modules teach parents cognitive behavioural strategies to apply in their child’s day-to-day life, including how to explore their child’s anxious thoughts, testing these thoughts by facing fears and problem-solving challenges, and the telephone sessions are designed to review progress and support parents to practise the strategies and problem solve any challenges. There is also an accompanying, optional game for mobile devices that is designed to help motivate the child to face their fears. We anticipate that one or two CWPs will support OSI delivery in this study. CWPs have received post-graduate training in the delivery of low-intensity psychological therapies for children and adolescents who experience difficulties with anxiety, low mood and behavioural problems. Study CWPs will receive regular supervision from clinical psychologists with expertise in treating childhood anxiety disorders.

### Outcomes

#### Feasibility outcomes and criteria for progressing to cluster randomised controlled trial

Feasibility outcomes related to negative impacts, acceptability, recruitment, and retention rates, and proposed clinical and health economic outcome measures, together with associated progression criteria are detailed in Table 1. To facilitate rapid progression to the main trial once the feasibility study is complete, interim criteria for progressing to the set-up phase for the cluster randomised controlled trial will be assessed once participant recruitment is complete, but while data collection is still ongoing. Once all data collection and required analyses are complete, the Study Steering Committee will then assess the full criteria for progressing to recruitment for the main trial. If there are no serious negative impacts or serious concerns about the acceptability of the procedures, at least 80% of the target population complete all assessments, and at least 12% of parents of children in study classes participate in OSI, we plan to progress to the randomised controlled trial and implement any indicated changes to the study procedures and outcome measures. If there are any serious harms or serious concerns about the acceptability of the study procedures, we will consult the Study Steering Committee about not progressing to the main trial. If the recruitment and retention rates are slightly below our targets (70–79% of the target population complete all assessments, and/or 9–11% of parents of children in study classes participate in OSI), we will consult the Study Steering Committee about progressing and consider whether changes to the protocol may improve recruitment/retention, and if recruitment/retention rates are markedly below our targets (< 70% of target population complete all assessments and/or < 9% of parents of children in study classes participate in OSI), we will consult the Study Steering Committee about not progressing to the main trial. However, as data collection is planned during the context of the COVID-19 pandemic, and associated restrictions (e.g. school closures, researchers unable to visit schools) may have an impact on recruitment/retention, the Study Steering Committee will consider this context when assessing the progression criteria related to recruitment and retention rates to determine whether continuing to the main trial is recommended or not.

**Table 1:** Feasibility outcomes and criteria for progressing to cluster randomised controlled trial

Feasibility outcome	Measure/method of assessment	Interim criteria for progressing to cluster randomised controlled trial set-up	Criteria for progressing to recruitment for cluster randomised controlled trial	Potential recommendations for cluster randomised controlled trial protocol
Negative impacts of the study procedures	• Monitoring participant reports throughout • Bespoke acceptability questionnaire (child-, parent-, teacher-report) • Qualitative interviews/discussion groups (children, parents/carers and school staff) • Study Steering Committee judgement	GO: Serious negative impacts have not arisen at this stage as a result of participation in the study procedures	GO: Serious negative impacts have not arisen as a result of participation in the study procedures	• Implement indicated changes to study procedures to minimise the risk of any negative impacts
		STOP: There are serious concerns about harms of study procedures, confirmed by Study Steering Committee	STOP: There are serious concerns about harms of study procedures, confirmed by Study Steering Committee	
Acceptability of all study procedures, including screening, feedback for parents/carers, brief intervention	• Monitoring participant reports throughout • Bespoke acceptability questionnaire (child-, parent-, teacher-report) • Qualitative interviews/discussion groups (children, parents/carers and school staff) • Study Steering Committee judgement	GO: No serious concerns have arisen about the acceptability of the study procedures	GO: No serious concerns about the acceptability of the study procedures	• Implement indicated changes to study procedures to minimise any concerns and maximise acceptability among children, parents/carers and school staff
		STOP: There are serious concerns about the acceptability of study procedures, confirmed by Study Steering Committee	STOP: There are serious concerns about the acceptability of study procedures, confirmed by Study Steering Committee	
Recruitment and retention rates	• Number (%) of eligible participants who complete baseline and follow-up assessments • Number (%) of participants who screen positive (target population) who complete baseline and follow-up assessments • Number (%) of participants who participate in OSI	GO: • At least 80% of participants who screen positive (target population) complete the baseline assessment. • At least 12% of parents of children in study classes^a^ to date participate in OSI	GO: • At least 80% of participants who screen positive (target population) complete all assessments. • At least 12% of parents of children in study classes^a^ participate in OSI	• Implement indicated changes to study procedures to improve recruitment/retention rates
		AMEND: • ≥ 70% and < 80% of participants who screen positive (target population) complete the baseline assessment. • ≥ 9% and < 12% of parents of children in study classes^a^ to date participate in OSI	AMEND: • ≥ 70% and < 80% of participants who screen positive (target population) complete all assessments. • ≥ 9% and < 12% of parents of children in study classes^a^ participate in OSI	
		STOP: • < 70% of participants who screen positive (target population) complete the baseline assessment. • < 9% of parents of children in study classes^a^ to date participate in OSI	STOP: • < 70% of participants who screen positive (target population) complete all assessments. • < 9% of parents of children in study classes^a^ participate in OSI	
Relevance and acceptability of all clinical and health economic outcome measures	• Proportion of missing data and patterns in missing responses/measures at baseline and follow-up assessments • Descriptive statistics for clinical and health economic outcomes • Qualitative interviews/discussion groups (children, parents/carers and school staff)			• Implement indicated changes to proposed outcome measures

*GO* = progress to cluster randomised controlled trial. *AMEND* = Consult Study Steering Committee regarding progression. *STOP* = Consult Study Steering Committee regarding not progressing

^a^Based on estimated 60% participation in parent-report screening; 20% screen positive

#### Child clinical outcome measures

##### Brief child anxiety screen

A 2-item parent-report questionnaire will be used to assess whether the child is experiencing problems with anxiety, and to identify the target population. The items assess the extent to which a child’s fears, worries or anxiety cause distress (Do your child’s fears, worries or anxiety upset or distress your child?) and interfere with family life (Do your child's fears, worries or anxiety make things difficult for your family as a whole?). Parents rate each item on a 4-point scale (No, not at all = 0; yes, only a little = 1, yes, quite a lot = 2; yes, a great deal = 3), and responses are summed to produce a total score. A cut-off score of ≥ 3 identifies children with anxiety disorders with 76% sensitivity and 80% specificity [17]. Children who score ≥ 3 out of 6 at baseline will be the target population, and total scores and screening outcomes (screen positive = score 3–6; screen negative = score 0–2) will be calculated at baseline and follow-up.

##### Child anxiety symptoms and interference

Child-, parent-, and teacher-report versions of the Brief Spence Children’s Anxiety Scale (SCAS-8-C/P/T) [20] and the child- and parent-report versions of the Revised Children’s Anxiety and Depression (RCADS)-Anxiety Scale will be used to assess anxiety disorder symptoms. The SCAS-8-C/P/T each consist of 8 items from the original SCAS [21, 22], and items are rated on a 4-point scale (0–3) that are summed to provide a total score. The SCAS-8 has the advantage of brevity, with evidence to support its ability to discriminate between children (aged 7–11) with anxiety disorders from a community sample [20]. The RCADS-C/P [23, 24] is widely used in clinical and community settings, and anxiety total scores reflect the sum of 37 anxiety items, each rated on a 4-point scale (0–3). Including these two alternative child anxiety symptom measures will provide the opportunity to assess the relevance and acceptability of both and inform our decision on whether to include one or both in the main trial. However, as neither the SCAS-8 nor the RCADS captures information about interference related to a child’s anxiety, we will also include additional items to assess anxiety-related interference for each reporter. Alongside the SCAS-8, parents will complete the 2-item brief anxiety screen detailed above, and children and teachers will complete the following corresponding items: children: Do fears or worries upset you?; Do fears or worries stop you from doing things?; Do your fears or worries make things difficult for people around you (e.g. family, friends, teachers)?; teachers: Do fears, worries or anxiety upset or distress this child?; Do this child's fears, worries or anxiety make things difficult for you or the class as a whole? Responses to these child- and teacher-report interference items will be summed to provide respective total scores (child: range 0–9; teacher: range 0–6).

##### Broader child clinical outcomes

Child low mood symptoms will be assessed using the child- and parent-report 10-item RCADS-Depression Scale (4-point rating scale (0–3); total score range 0–30). The well-established child- and parent-report Strengths and Difficulties Questionnaire (SDQ-C/P) [25, 26] will be used to measure broader emotional and behavioural symptoms, providing a Total Difficulties score (range 0–40), as well as subscales scores (emotional problems [range 0–10]; Conduct problems [range 0–10]; Peer problems scale [range 0-10]; Hyperactivity [range 0–10]).

#### Health economic outcomes and measures

##### Quality of life

Child- and parent-report versions of the Child Health Utility-9D (CHU-9D) [27, 28] and the EQ-5D-Y [29] will be used to assess children’s quality of life. The CHU-9D is a preference-based measure of health-related quality of life which allows the calculation of Quality Adjusted Life Years (QALYs) for use in cost utility analysis. It includes nine dimensions (worried, sad, pain, tired, annoyed, schoolwork, sleep, daily routine, activities) each with five ordered response levels. The measure was originally developed and validated with children aged 7–11 years. The EQ-5D-Y is a child-friendly version of the EQ-5D that was introduced by the EuroQol Group in 2009 as a comprehensible instrument suitable for measuring health-related quality of life of children and adolescents aged 8–15 years. It includes five dimensions (mobility; looking after myself; doing usual activities; having pain or discomfort; and feeling worried, sad or unhappy) each with three ordered response levels. It can be used to derive QALYs. There is no clear standard when it comes to measuring Health-related Quality of Life Instruments for children and young people, but the CHU-9D and the EQ-5D-Y are among the most used instruments. We will therefore use both in this study to inform our choice for the main trial.

To measure parents’ quality of life, we will use the EQ-ED-5L [30]. The EQ-5D-5L is a well-validated preference-based measure of health-related quality of life in adult populations, designed to estimate quality adjusted life years (QALYs) and widely used across disease areas. It includes five dimensions covering domains of everyday life, i.e., mobility, self-care, usual activities, pain/discomfort, and anxiety/depression, each with five ordered levels of response.

##### Resource use

A societal perspective for resource use will be adopted in recognition of the fact that economic costs of mental health have wide consequences beyond the health and social care sectors, including education and the labour market. We will use a modified version of the Client Service Receipt Inventory (CSRI) [31] form and therapist, supervisor and school staff logs to identify and measure: (i) resources used in delivering the iCATS-i2i screening, feedback, and intervention procedures; (ii) child and parent individual resource use data including health and social care system and other sector resources (e.g. GP use, referrals, child and adolescents mental health services, educational services); (iii) other child-, family-, society-borne resource use, including child time off school, parent time spent related to child anxiety problems, including time off work (i.e. productivity losses for the wider economy). Parents will complete the modified CSRI at baseline (with reference to the previous 3 months) and follow-up (with reference to the study period). At baseline, parents will also be provided with a diary to keep a record of service use and time off work/school, to facilitate answering the questions at follow-up. School staff will be asked to complete a log throughout the study to record time spent on study activities (e.g. administering questionnaires, distributing information to families), and study therapists and supervisors will complete logs to record time spent on activities related to delivering OSI.

#### Additional information and measures

##### Socio-demographic information

Parents will provide socio-demographic information about their child (date of birth, gender, ethnicity, eligibility for free school meals), themselves (age, gender, ethnicity, relationship to child, whether they have a partner), and their household (parent highest level of education, parent employment status, parent occupation, income, postcode, housing tenure, number of children living in household). Child demographic information will also be collected from the child’s school records (gender, ethnicity, eligibility for free school meals, any special education needs). Teachers will provide some background information about themselves (age, gender, role, number of years teaching experience), and school-level demographic characteristics (local education authority area, number of pupils on the roll, percentage of pupils eligible for free school meals, percentage of pupils on special educational needs support, percentage of pupils with English as an additional language) will be collected from the Department for Education public records.

##### Acceptability questionnaire

A bespoke child-, parent-, and teacher-report questionnaire measure will be used at follow-up to assess acceptability of study procedures. Participants rate the extent to which they agree with statements about their experiences of completing questionnaires, feedback following screening, and (where applicable) OSI, with space to provide additional written feedback. Items address both negative and positive experiences of study procedures.

##### School attendance and punctuality and learning information

Schools will be asked to provide information on children’s attendance, punctuality, and learning outcome to inform our choice about how best to collect this information in the main trial.

##### Measures collected through OSI

Parents will complete weekly questionnaires via the OSI website to guide the intervention. Questionnaires include measures of child anxiety symptoms (RCADS/tracked RCADS-subscale, SCAS-8), interference related to the child’s anxiety (Child Anxiety Impact Scale; CAIS/CAIS-global subscale [32, 33], overall functioning (Outcome Rating Scale; ORS [34]), progress towards meeting intervention goals (Goal Based Outcomes; GBO [35]), and the therapeutic relationship (Session Rating Scale; SRS [36]). OSI usage data (e.g. modules completed, optional interactive activities completed) will also be collected.

##### Qualitative interviews/discussion groups

Interviews/discussions will be topic-guided and explore participants’ experiences of iCATS-i2i procedures, including any negative experiences or concerns, and views of meaningful outcomes. Interview guides will be tailored for each participant group (children, parents, school staff) (see Additional file 3 for indicative interview guides). Interviews/discussion will be audio-recorded and transcribed, with identifiable information removed at the point of transcription.

### Data management

REDCap (Research Electronic Data Capture) databases will be used to capture data via online surveys and paper questionnaires directly inputted by researchers. Data captured will be held on University of Oxford servers and access will be restricted to the study team members. Once data collection is complete, data will be permanently deleted from REDCap and stored on a restricted access folder on the University of Oxford network.

Schools and participants will be assigned unique IDs, and these IDs will be used to label all study data. A document linking ID to names/contact information will be stored on a restricted access folder on the University of Oxford network. This linking document and other personally identifiable information (consent/assent, audio recordings) will be stored for as long as needed for research purposes and appropriate safeguards are in place, and then permanently deleted.

Pseudonymised study data will be shared with study statisticians and health economists for analysis, via the University of Oxford’s OneDriveforBusiness.

### Sample size

We aim to recruit approximately 360 children from 6 primary/junior schools (30 children per class, two classes per school), and their parents and class teachers. This sample size will allow us to assess feasibility outcomes in schools with varied geographic and demographic profiles, and will provide a sufficient number of families with varied characteristics to identify any issues with acceptability or feasibility. In line with a recent primary school-based trial in the UK [19], we expect to collect parent baseline measures from approximately 60% of children in participating classes (*n* = 216), and on the basis of findings in our measurement development study [17], we expect approximately 20% of these will screen positive (target population: *n* = 43).

### Planned data analysis

#### Statistical analysis

Participant flow will be presented using a CONSORT diagram. Recruitment and retention percentages and the percentage of missing data will be presented for each reporter at baseline and follow-up with 95% confidence intervals. Recruitment and retention rates, and OSI uptake will be presented for the target population (children who screen positive for anxiety problems at baseline), and the wider group of all children in participating classes. The nature of any missing data will be explored, and the characteristics of children/parents/teachers who did and did not respond to the various questions/measures will be compared. Clinical and economic outcomes will be summarised using means and standard deviations or numbers and percentages. Suitability and acceptability of the measures for the main trial will be assessed on the basis of both rates of responses and from participant feedback.

### Qualitative analysis

Transcripts of the qualitative data will be analysed using an adapted form of Template Analysis [37]. The preliminary template will be structured by categories identified in the co-design work [13], and transcripts will initially be coded using this preliminary structure. The template will then be developed further to incorporate additional codes identified by preliminary coding of the data. The aim of the analysis will be to identify any issues related to acceptability or feasibility of the iCATS-i2i procedures and OSI. Credibility of the qualitative analysis will be checked via analytic triangulation through reflective discussions with supervisors and co-analysts. Broader credibility-checking for the identified acceptability and feasibility issues will take place within a small expert reference group (including parent and school representatives) prior to informing the subsequent main trial.

### Monitoring

The PI (CC) and Study Lead (TR) will supervise the day-to-day running of the study and researchers involved in data collection activities. The Study Management Group (SMG) includes the PI and co-investigators (including school and parent representatives). The SMG will oversee and consult on all aspects of the study and dissemination of findings, meeting at least twice a year and with subgroup discussions throughout. An independent Study Steering Committee (SSC) including members with expertise in evaluations of school-based mental health interventions, statistical and qualitative methods, and school leadership, will meet at least twice during the study. The SSC will monitor study progress, advise on management and scientific issues, and ensure there are no adverse events or substantial deviations from study protocol. As detailed above, the SSC will review progression criteria and make recommendations about continuing to a subsequent cluster randomised controlled trial.

## Discussion

This study aims to establish the feasibility of a subsequent cluster randomised controlled trial to evaluate procedures for identifying and supporting children with anxiety problems through primary schools. If findings support progression and subsequent implementation in primary schools, iCATS-i2i procedures will improve children’s access to effective, early intervention for anxiety difficulties through schools. We also hope that our findings will inform the development and evaluation of broader school-based approaches to identifying and supporting children and adolescents with mental health difficulties in primary and secondary school settings.

It is important to acknowledge some practical and methodological limitations with this study. Firstly, this study will be conducted in the context of the COVID-19 pandemic, and although we will adjust procedures as needed (e.g. school staff rather than researchers administer questionnaires with children), we will need to consider this context and the impact of any adjustments or disruptions when interpreting findings. For example, it is possible that children’s anxiety, the relevance of questionnaire items, and recruitment rates may vary depending on COVID-19 restrictions. Secondly, although we are using an initial ‘opt-out’ approach to children’s involvement and we will make the intervention available to all families in participating classes, there may be children who might benefit from support with anxiety problems who we do not reach (e.g. children who are not regularly attending school). In particular, the fact that we will only be able to provide feedback on screening responses and contact parents by telephone/email when parents complete the screening questionnaire and/or provide us with their contact details means that we may miss families in challenging situations that may impede their participation. We will explore potential barriers to parental involvement at all stages of the study in our qualitative interviews with school staff and parents, and implement any indicated changes to minimise such barriers ahead of a main trial.

## Supplementary Information

**Additional file 1.** SPIRIT 2013 Checklist: Recommended items to address in a clinical trial protocol and related documents*.

**Additional file 2.** Schedule of enrolment, intervention and assessment.

**Additional file 3.** Indicative interview topic guides.

## Data Availability

Datasets and study materials generated during the current study will be made available in a public repository.

## Abbreviations

iCATS-i2i: Identifying Child Anxiety Through Schools-identification to intervention
OSI: Online Support and Intervention for child anxiety
SPIRIT: Standard Protocol Items: Recommendations for Interventional Trials
CWP: Children’s Wellbeing Practitioner
SCAS: Spence Children’s Anxiety Scale
RCADS: Revised Children’s Anxiety and Depression Scale
SDQ: Strength and Difficulties Questionnaire
CHU-9D: Child Health Utility 9 Dimension instrument
EQ-5D-Y: EuroQol 5 Dimension -Youth version instrument
EQ-5D-5L: EuroQol 5 Dimension 5 Level instrument
CSRI: Client Service Receipt Inventory
CAIS: Child Impact Anxiety Scale
ORS: Outcome Rating Scale
SRS: Session Rating Scale
GBO: Goal Based Outcomes
SSC: Study Steering Committee
SMG: Study Management Group
PI: Principal Investigator
REDCap: Research Electronic Data Capture
