# Supplementary material for: Identifying Child Anxiety Through Schools-identification to intervention (iCATS-i2i): protocol for single-arm feasibility trial
Source: Pilot Feasibility Stud. 2022 Aug 10;8:176. doi: 10.1186/s40814-022-01140-x (PMC9363860; doi:10.1186/s40814-022-01140-x)
Supplement: Supplementary file 2 — Additional file 2. Schedule of enrolment, intervention and assessment. [file 40814_2022_1140_MOESM2_ESM.docx]

Figure 2 Schedule of enrolment, intervention and assessment

|  | **Study period** | | | | |
| --- | --- | --- | --- | --- | --- |
|  | **School enrolment** | **Baseline**  **Week 0-2** | **Parent/carer feedback**  **Week 3-4** | **OSI**  **Week 4-12** | **Follow-up**  **Week 12** |
| **Enrolment** | | | | | |
| School consent | X |  |  |  |  |
| Parent opt-out |  | X |  |  |  |
| Child assent |  | X |  |  |  |
| Parent consent |  | X | X (if not provided at baseline) |  |  |
| Teacher consent |  | X |  |  |  |
| Qualitative interview/discussion group consent/assent |  |  |  | (if not provided previously) | |
| **Feedback and Intervention** | | | | | |
| Written and telephone feedback for parents/carers |  |  | X |  |  |
| OSI |  |  |  |  |  |
| Anxiety lesson |  |  |  |  |  |
| **Assessments** | | | | | |
| **Child anxiety screen** | | | | | |
| 2-item child-anxiety screen (parent-report) |  | X |  |  | X |
| **Demographic and contact information** | | | | | |
| School level demographic information (DfE website) | X |  |  |  |  |
| Child demographic information from school records |  | X |  |  |  |
| Family demographic information (parent-report) |  | X | X (if not provided at baseline) |  |  |
| Teacher demographic information |  | X |  |  |  |
| Qualitative interview/discussion group participant demographic information |  |  |  | (if not provided previously) | |
| Family contact information |  | X | X (if not provided at baseline) |  |  |

| **Broader child clinical outcomes** | | | | | |
| --- | --- | --- | --- | --- | --- |
| Brief child anxiety questionnaire (child-report, teacher report) |  | X |  |  | X |
| SCAS-8 (child- report, parent-report, teacher report) |  | X |  |  | X |
| RCADS-anxiety scale (child-report and parent-report) |  | X |  |  | X |
| RCADS-depression scale (child-report and parent-report) |  | X |  |  | X |
| SDQ (child-report and parent-report) |  | X |  |  | X |
| **To guide OSI** | | | | | |
| RCADS (parent-report) |  |  |  | OSI module 0, 6, follow up |  |
| RCADS-target subscale (parent- report) |  |  |  | OSI module 1,2, 3, 4, 5 |  |
| CAIS (parent- report) |  |  |  | OSI module 0, 6, follow up |  |
| CAIS-overall subscale (parent- report) |  |  |  | OSI module 1,2, 3, 4, 5 |  |
| SCAS-8 (parent- report) |  |  |  | OSI module 0, 1, 2, 3, 4, 5, 6, follow up |  |
| Goal-based outcome (parent- report) |  |  |  | OSI module 2, 3, 4, 5, 6, follow up |  |
| Outcome Rating Scale (parent- report) |  |  |  | OSI module 0, 1, 2, 3, 4, 5, 6, follow up |  |
| Session Rating Scale (parent-report) |  |  |  | OSI module 0, 1, 2, 3, 4, 5, 6, follow up |  |
| **Health economic measures and outcomes** | | | | | |
| CHU-9D (child-report and parent-report) |  | X |  |  | X |
| EQ-5D-Y (child- report and parent-report) |  | X |  |  | X |
| EQ-5D-5L (parent-self-report) |  | X |  |  | X |
| Adapted client services receipt inventory (parent-report) |  | X |  |  | X |
| Client services diary (parent-report) |  | X |  |  |  |
| Wellbeing practitioner log |  |  |  |  |  |
| Supervisor log |  |  |  |  |  |
| School staff log (time spent on study activities) |  |  |  |  |  |

| **Additional measures and information** | | | | | |
| --- | --- | --- | --- | --- | --- |
| Child attendance, punctuality, academic progress from school records |  | X |  |  | X |
| Bespoke acceptability questionnaire (child- report, parent-report, teacher-report) |  |  |  |  | X |
| OSI usage data |  |  |  |  |  |
| Qualitative interviews/discussion groups (child, parent, school staff) |  |  |  |  |  |

*Note.* DfE=Department for Education; RCADS=Revised Child Anxiety and Depression Scale; SCAS-8: Brief Spence Children’s Anxiety Scale; CAIS=Child Anxiety Impact Scale; CHU-9D=Child Health Utility-9D; EQ-5D-Y=EuroQuality of life-5 level instrument-Youth version; EQ-5D-L=EuroQuality of life-5 level instrument; OSI=Online Support and Intervention for child anxiety
