# Supplementary material for: Identifying Child Anxiety Through Schools-identification to intervention (iCATS-i2i): protocol for single-arm feasibility trial
Source: Pilot Feasibility Stud. 2022 Aug 10;8:176. doi: 10.1186/s40814-022-01140-x (PMC9363860; doi:10.1186/s40814-022-01140-x)
Supplement: Supplementary file 3 — Additional file 3. Indicative interview topic guides. [file 40814_2022_1140_MOESM3_ESM.docx]

**Additional file 2: Indicative interview topic guides**

**Indicative guide for in-depth interviews and discussion groups with children**

**Introduction**

- Researcher/s introduce the aim of the research and the discussion group/interview.
- Researcher/s summarise key activities (questionnaires, feedback, online intervention)

**Issues to explore in the interview/discussion**

• What did you think about the iCATS assembly where iCATS was first introduced?

• Was there any more information you would have liked to know about iCATS before joining in?

- What did you think about the iCATS class lesson on fears and worries?
  - What bits did you like about the lesson? What bits did you not like?

• What did you think about filling in the questionnaire about your fears and worries?

- Did you do the questionnaire at home or at school?
- Would you have preferred to fill in the questionnaire at home with your parent(s) or at school?
- Did you do the questionnaire on paper or on the electronic tablet?
- Could anything have been done differently to make filling in the questionnaire easier for you?
- What did you think about your parent(s) doing the lessons to help you with your fears and worries?
- How did doing the iCATS activities with your parent(s) make you feel?
  - Were there any activities you found really fun?
  - Were any activities hard?
  - Why do you think that was?
- How did you find using the Monster's Journey game?
- Did you speak to anyone (e.g. friends, family, teachers) about your parents doing the lessons to help you with your fears and worries?
  - What did you say? How did they respond?
  - If you didn’t speak to anyone, why was this?
- Do you think your parents doing the lessons about your fears and worries had any effect on other members of your family or how your family gets along?
  - Why or why not?
- Is there any extra help for your fears or worries that you would have liked to have?
- Are there any other thoughts you have about the iCATS project that we should know?

**Indicative guide for in-depth interviews and discussion groups with parents**

**Introduction**

- Researcher/s introduce the aim of the research and the discussion group.
- Researcher/s summarise key activities (questionnaires, feedback, online intervention)
- Researcher confirms what stage of iCATS family is at (or where they dropped out)

**Issues to explore in the interview**

- 1. How have you found taking part in the iCATS study so far?
- How did you hear about the project?
- What made you want to get involved?
- Were there any issues or concerns you were hoping iCATS would help with?
- What did you hope to get out of taking part in iCATS?
- Did you have any concerns or worries that made you hesitant to get involved?
- Was there anything you think could have been done to encourage you/others to get involved?

2. How did you get on with the screening questionnaires and consent forms?

- Did you fill these in or did your child’s other parent? Why was this?
- What did you feel about completing your questionnaires, particularly those that asked about your own feelings?
- Was there anything that you found difficult in filling in the questionnaires?
- Was there anything that could’ve been made easier for you here?
- How did you find accessing these online (or by paper)?
- How did your child get on with these?
- Did they do the questionnaire at home with you or at school? What did you think about this approach?
- (if applicable) – did they need any help from you to fill them in? Why or why not?
- How did you feel about your child’s teacher also filling in a questionnaire about your child?
- What are your thoughts about the questionnaires and your information being completed online?
- Would you be more comfortable with paper copies managed by your child’s school?
- Was there anything that you feel could have been done differently here?
- We've found that the parent questionnaires are the most accurate for determining whether a child has likely anxiety difficulties. How do you feel about our just using the parent questionnaires to inform our feedback we give to families?
- Was there anything you feel you or your child gained or learnt from filling in the questionnaires?

3. How did you find the **feedback** about your child’s score?

- What did you think of the feedback letter?
- Was the outcome what you expected?
- How did you find the telephone feedback call?
- Initially we planned for the school iCATS lead to give this feedback, how did you find the feedback coming from the research team instead?
- Did you have any concerns at this stage?
- What did you think about the things that were said in the feedback call and letter?
- How did the feedback you got on the questionnaires affect how you felt about doing the OSI intervention?
- Prompt for screen positives- if you hadn’t been told that the scores indicated possible anxiety difficulties do you think you would have taken up OSI? Why or why not?
- Prompt for screen negatives- did being told that your child hadn’t screened as probably having anxiety difficulties affect how you felt about doing the OSI intervention?
- Could anything have been done differently here?
- Was there any more information you would have liked to have had?

4. How did you find **accessing OSI**?

- What did you think of the resource and exercises?
- What did you think about everything being online/remote?
- How do you think this compares to a F2F course?
- How did you find doing the activities with your child?
- What impact do you think the activities have had on their fears and worries?
- How do you feel about managing your child’s difficulties with fears and worries having done OSI?
- Has your knowledge or confidence in supporting your child changed following OSI?
- Has there been any change in your family life since taking up OSI?
- Have you become aware of any new sources of support as a result of being part of OSI?
- What did you think of the weekly phone calls?
- How do you feel about the number or length of sessions? Were there enough or too many or just right?
- How have you found the 1 month break? OR How *do* you feel about there being a 1 month break?
- How do you feel about your child’s ‘discharge plan’?
- Will you share this with your child’s school? Why or why not?
- In an ideal world, is there any other support or help you would’ve liked to receive?
- Could anything have been made easier for you/others to keep engaging with OSI?
- How did you feel about providing your child’s GP details?

5. Have you spoken to or interacted with your child’s school about iCATS?

- What was this experience like?
- Could anything have been improved here?

6. Have you spoken with other people about iCATS?

- Have you spoken to any parents who dropped out of or chose not to take part in iCATS? Do you know why they made this decision?
- After finishing OSI do you think you will speak to other people about it?

7. Is there anything we can do to make sure iCATS works well for other families in future?

- How would you describe your child’s school culture or attitude towards mental health or anxiety?
- Do you think iCATS may have any broader effects on your child’s school or your community?

**Issues to explore in the interview for those who dropped out**

- At what stage did you begin to feel like iCATS wasn’t suitable for you?
- Do you think your experiences of CV-19 had any impact on this decision?
- Was there anything you didn’t feel you were getting from the iCATS / OSI project that could be improved?
- How does your child feel about not being involved in the project anymore?
- Did you have any particular needs you didn’t feel were addressed by iCATS/OSI?
- Did you access the online resources (pdf) instead?
  - Why or why not?

**Indicative guide for in-depth interviews and discussion groups with teachers**

**Introduction**

- Researcher/s introduce the aim of the research and the discussion group.
- Researcher/s summarise key activities (questionnaires, feedback, online intervention)
- Researcher confirms what stage of iCATS school is at (or where they dropped out)

**Issues to explore in the interview**

1. How have you found being part of the iCATS study so far?

- How did you hear about the project?
- What made you and your school want to get involved?
- Were there any particular motivators for your school to want to join in? What were these?
- Are there any factors that made you or your school hesitant to take part? (e.g. demands on staff time, screening/intervention programme could be upsetting, etc)
- Was there anything you think could have been done to encourage your school (and other schools) to get involved?
- What did you hope you/your pupils would get out of taking part in iCATS?
- How did you get on with the screening questionnaires and consent forms?
- Was there anything that could’ve been made easier for you here?

2. What did you think about filling in the **questionnaires** about your pupils?

- What did you think about the number/length of questionnaires?
- How did you find accessing these online?
- How did your pupils get on with these?
- Did they do the questionnaire at home or at school? What did you think about this approach?
- (if applicable) – did they need any help from you to fill them in? Why or why not?
- Was there anything that you feel could have been done differently here?
- Do you know how any of your class's parents got on with filling in their questionnaires?
- Did any parents have any difficulties accessing or filling them in?
- Why may some parents have a tough time filling in the questionnaires?
- Was there anything we could do to support parents better during this process?
- How do you feel about just using parent questionnaires to inform feedback?
- Is there is anything you feel you learned from filling in questionnaires?
- Did filling in the questionnaires have any impact on your knowledge or confidence in supporting children in your class?

3. How did you find the **feedback** about pupils’ scores?

- What did you think of the feedback letter? Did you see a copy?
- Were the outcomes what you were expecting?
- Initially we planned for the school iCATS lead to give this feedback, how do you feel about the feedback coming from the research team instead?
- Did you have any concerns at this stage?
- Could anything have been done differently here?
- Was there any more information you would have liked to have had?

4. Have you spoken to or interacted with your pupils or parents about their experience of iCATS?

- What was this experience like?
- Did you get asked any questions by pupils/parents? How did this go?
- Did you speak with any parents/pupils who didn’t want to take part or dropped out? What seemed to contribute towards this?
- Could anything have been improved here?
- Have you spoken with other people about iCATS? (e.g. colleagues, your own friends/family)

5. How would you describe your school’s culture or attitude towards mental health or anxiety?

- What is the attitude towards mental health in the local community?
- Do you think iCATS has had or may have any broader effects on your school or your community?

6. Is there anything we can do to make sure iCATS works well for other schools or families in future?
